# Efficacy of structured psychoeducational group therapy grounded in Yalom’s interpersonal principles for parents of adolescents with depression

**DOI:** 10.3389/fpsyt.2026.1896423

**Published:** 2026-08-21

**Authors:** Tongtong Wang, Qiyong Li, Tao Ding, Songying Fang, Menglan Zu, Yanli Zhao, Lin Ma

**Affiliations:** 1 Peking University Huilongguan Clinical Medical School, Beijing Huilongguan Hospital, Capital Medical University, Beijing, China; 2 Beijing Haidian District Mental Health Prevention and Cure Hospital, Beijing, China

**Keywords:** adolescent depression, family function, parents, structured psychoeducational group therapy, Yalom’s interpersonal principles

## Abstract

**Objective:**

To explore the efficacy of structured psychoeducational group therapy grounded in Yalom’s interpersonal therapeutic principles on parental anxiety, depressive symptoms, and family cohesion and adaptability among parents of adolescents with depression, and to provide clinical evidence for parent-focused family psychological support for adolescents with depression.

**Methods:**

A total of 90 parents (45 couples) of adolescents aged 12–18 with depression were recruited from the outpatient department of a psychiatric hospital in Beijing between June 2023 and June 2024. Both parents of each adolescent were required to participate. Randomization was performed at the family level, with participants randomly assigned to an intervention group or a control group in a 1:1 ratio using sealed envelope allocation concealment. The intervention group received 12 weekly 90-minute sessions of structured psychoeducational group therapy grounded in Yalom’s interpersonal therapeutic principles, with standardized themed modules including peer support, emotional catharsis, interpersonal reflection, cognitive restructuring, and family communication skill training; the control group received no systematic psychological intervention. All analyses were conducted on a completer basis. Assessments were conducted at pre-intervention, post-intervention, and 3-month follow-up using the Self-Rating Anxiety Scale (SAS), Self-Rating Depression Scale (SDS), and Family Adaptability and Cohesion Scale (FACES II). A 2×3 mixed-design repeated-measures ANOVA was applied to compare intervention effects, with partial eta-squared (*η²*) reported as the measure of effect size.

**Results:**

Significant main effects of time were observed for anxiety, family cohesion, and family adaptability (all *P* < 0.05), while the main effect of time for depression was not significant (*P*>0.05). A significant group-by-time interaction was found for family cohesion (*F* = 4.487, *P* = 0.018, *η²* = 0.049), interactions for family adaptability, anxiety and depression were not significant (all *P*>0.05). The intervention group showed sustained reductions in anxiety and gradual improvements in family cohesion and adaptability over the study period. No statistically significant changes in any outcome were observed in the control group across time points.

**Conclusion:**

This study provides preliminary evidence for the feasibility and potential benefits of short-term structured psychoeducational group therapy grounded in Yalom’s interpersonal therapeutic principles among parents of adolescents with depression, particularly for improving family cohesion. Intervention-specific effects on parental anxiety, depression, and family adaptability were not statistically significant, and further research with active control conditions and larger samples is warranted.

## 1 Introduction

According to authoritative data in 2021, depression affects more than 350 million people worldwide and approximately 54 million people in China, with the highest prevalence among young people.

A Malaysian study found that 26.2% of secondary school students met the criteria for depressive symptoms (1). In Germany, the detection rate of depressive symptoms among adolescents aged 12–17 was 8.2% (2). In the United States, the prevalence of depression among adolescents increased from 8.7% in 2005 to 11.3% in 2014 (3). In Japan, the rate of depressive symptoms among junior high school students was 24.9% (4). In South Korea, nearly 13.6% of adolescents reported recent depressive symptoms (5). In Canada, the prevalence among secondary school students was 41.4% (6). A Chinese meta-analysis of 51 epidemiological studies reported a pooled prevalence of depressive symptoms among secondary school students of 24.3% (7).

Against this backdrop of high global prevalence of adolescent depressive symptoms, the family stands as a foundational developmental context with well-documented influences on the onset, severity, and prognosis of youth depression. Grounded in Bowen’s family systems theory, which posits that family members exert reciprocal, systemic influences on one another’s psychological functioning (8), a substantial body of empirical work has linked family relationship quality to adolescent depressive outcomes. Convergent evidence from Chinese studies indicates that harmonious family dynamics and sufficient parental care function as protective factors, while family dysfunction and inadequate parental support may represent major triggers for adolescent depression (9–12). At a broader quantitative level, a recent three-level meta-analysis of 18, 283 participants corroborates these findings, documenting a robust negative association between perceived family resilience and symptoms of anxiety and depression (r = −0.31) and confirming the protective role of family adaptive capacity against adolescent mental health impairment (13).

Moving beyond broad associations to specific interactional mechanisms, observational and ecological momentary assessment research has unpacked how day-to-day parenting behaviors shape adolescent affective outcomes. Observed parental psychological control has been associated with poorer affective states in adolescents with depression, whereas supportive parenting broadly fosters emotional well-being—though adolescents with depression appear to exhibit a negativity bias in their retrospective perceptions of parental behavior (14, 15). A multimethod, multi-informant intensive longitudinal study further strengthens this evidence base, demonstrating that supportive parent–youth communication serves as a core family protective mechanism for adolescent depression and highlighting the clinical utility of targeting daily family interaction patterns in intervention development (16). Taken together, this convergence of theoretical frameworks, meta-analytic evidence, and fine-grained interactional research positions the family system as a critical, modifiable leverage point for addressing adolescent depression.

Numerous studies show that parental anxiety and depression can influence adolescent anxiety and depression (17–19). Twin and molecular genetic studies confirm genetic contributions to adolescent depression (20). The Theory of Primitive Emotional Contagion posits that emotions synchronize through unconscious mimicry and feedback mechanisms (21). When adolescents perceive parental negative emotions, they unconsciously mimic them, activating corresponding neural patterns and inducing emotional contagion (22). Conversely, strong family cohesion, warm emotional climate, and parental emotional support are protective factors against depression (23).

Integrating family systems theory and emotional contagion theory, the current study proposes that intervening on parental emotional well-being and family interaction patterns may disrupt the intergenerational transmission of depressive symptoms and enhance family resilience. A structured psychoeducational group approach grounded in Yalom’s interpersonal therapeutic principles provides a well-suited framework for this goal. As a social microcosm, the group mirrors real-world family and interpersonal dynamics, allowing members to identify maladaptive interaction patterns, receive peer feedback, and practice new communication skills within a supportive environment (24). Unlike unstructured process-oriented Yalomian groups that follow spontaneous in-the-moment dynamics, the present intervention adopts a manualized, theme-based design tailored to the specific needs of parents of adolescents with depression. The therapeutic factors of universality, altruism, interpersonal learning, and catharsis are hypothesized to reduce parental caregiving distress and improve family functioning, which in turn may support adolescent recovery.

As role models and primary family influencers, parents profoundly shape the onset, treatment, recovery, and prognosis of adolescent depression. Prevention and treatment of adolescent depression must be accompanied by parental education and intervention.

Group therapy and family interventions improve family function and reduce caregiver distress (25–28). Psychoeducational group interventions informed by Yalom’s interpersonal principles emphasizes interpersonal interaction, peer support, emotional catharsis, and existential reflection, viewing the group as a social microcosm that enables members to recognize and modify maladaptive interpersonal patterns. It has the dual functions of emotional support and cognitive reconstruction.

Existing domestic and international interventions for parents of adolescents with depression primarily use health education or supportive discussions (29, 30), or multifamily therapy formats (31). Structured, standardized short-term interventions grounded in Yalom’s interpersonal theory remain scarce, and long-term effects on parental mood and family function are unclear.

This study tested the following primary and secondary hypotheses:

1. Primary hypothesis: Parents in the intervention group will show significantly greater improvements in family cohesion from baseline to 3-month follow-up compared with parents in the control group.
2. Secondary hypotheses:

- Parents in the intervention group will show significantly greater reductions in anxiety and depressive symptoms compared with controls.

- Parents in the intervention group will show significantly greater improvements in family adaptability compared with controls.

This study adopted a randomized controlled design and implemented a 12-session manualized psychoeducational group intervention grounded in Yalom’s interpersonal therapeutic principles requiring both parents of adolescents with depression to participate, aiming to maximize improvements in the family support system. We conducted 3-month longitudinal follow-up to examine intervention effects on parental anxiety, depression, and family function, providing evidence for scalable family interventions.

## 2 Methods

### 2.1 Participants

Parents of adolescents aged 12–18 with depression attending the outpatient department of Beijing Huilongguan Hospital between June 2023 and June 2024 were recruited.

Inclusion criteria:

(1) Adolescents met ICD-10 criteria for depressive episode. With diagnoses confirmed by attending psychiatrists via structured clinical interview and medical record review. (2) Parents provided written informed consent. (3) No severe physical or mental illness, able to complete assessments and intervention.

Exclusion criteria:

(1) Receiving concurrent systematic psychotherapy. (2) Adolescents with severe physical illness or psychosis. (3) Parents with cognitive or communication impairment.

A total of 100 parents (50 families) were randomized 1:1 into intervention and control groups. Randomization was performed at the family level using SPSS-generated random numbers, with sealed envelope allocation concealment to ensure allocation sequence concealment. The intervention group included 50 parents (25 families), and the control group 50 parents (25 families). Five families in the control group were lost to follow-up; the final analytical sample was 90 participants (50 in intervention, 40 in control). The study was approved by the Ethics Committee of Beijing Huilongguan Hospital (No. 2023-66). All participants provided written informed consent. The study was conducted in full compliance with ethical principles for human subjects research.

### 2.2 Sample size calculation

Sample size was estimated using G*Power 3.1 for the primary group-by-time interaction effect in a 2 (group) × 3 (time) mixed-design repeated-measures ANOVA. The following *a priori* assumptions were specified: a medium effect size of f = 0.25 (based on Cohen’s conventions), a two-tailed α level of 0.05, a statistical power (1−β) of 0.80, three measurement time points, and a correlation of 0.5 between repeated measures (consistent with previous similar psychosocial intervention studies). The calculation yielded a required total sample size of 86 participants. The final enrolled sample of 90 parents exceeded this requirement, providing sufficient statistical power to detect the hypothesized interaction effect.

### 2.3 Study design

A 2 (group: intervention/control) × 3 (time: pre-intervention/post-intervention/3-month follow-up) mixed design was adopted. The intervention group received 12 sessions of structured psychoeducational group therapy grounded in Yalom’s interpersonal therapeutic principles; the control group received no systematic psychological intervention. Adolescents in both groups continued routine outpatient treatment and follow-up.

#### 2.3.1 Randomization and blinding

Randomization was conducted at the family level using SPSS-generated random numbers, with sealed envelope allocation concealment to prevent selection bias.

Blinding: Outcome assessors were blinded to group assignment; participants and therapists were unblinded due to the nature of the psychosocial intervention.

#### 2.3.2 Intervention: structured psychoeducational group grounded in Yalom’s interpersonal therapeutic principles

The intervention was a manualized, structured psychoeducational group program informed by core therapeutic principles from Yalom’s interpersonal theory, including universality, interpersonal learning, catharsis, and group cohesion. It differs from traditional unstructured process-focused Yalomian groups in that it follows a fixed 12-session thematic curriculum with standardized exercises, designed specifically for the homogeneous population of parents of adolescents with depression.

Therapists: Two intermediate-level psychotherapists with ≥5 years of group psychotherapy experience, who completed standardized training in Yalom’s interpersonal group principles and the structured intervention manual prior to study initiation. Weekly group supervision was provided by a senior group psychotherapist throughout the study to ensure treatment fidelity. Treatment adherence was rated via session audio recordings by an independent rater, with an overall fidelity score of 92% across all sessions.

Group setup: One group of 10 parents (5 couples), with 5 parallel groups run concurrently. Weekly sessions, 90 minutes each, for 12 weeks total.

The intervention was structured around core Yalom therapeutic factors, operationalized as follows across the 12 sessions:

- Sessions 1–2 (Group Formation): Warm-up activities, member introductions, group norm establishment, and shared discussion of caregiving stress, designed to build group safety and foster universality.
- Sessions 3–4 (Emotional Awareness and Validation): Structured sharing activities and peer feedback to normalize caregiving distress, reduce stigma, and facilitate emotional expression, targeting catharsis and group cohesion.
- Sessions 5–6 (Parent-Child Interaction Reflection): Guided discussion of parent-child relationship patterns and communication challenges, to promote awareness of maladaptive interaction styles.
- Sessions 7–8 (Interpersonal Learning and Cognitive Restructuring): Structured interpersonal feedback exercises and exploration of family intergenerational patterns, to support cognitive restructuring and recognition of maladaptive parenting patterns.
- Sessions 9–10 (Family Communication Skill Building): Experiential exercises (e.g., Satir communication stance work) and role-play to practice adaptive family communication and collaborative parenting.
- Session 11 (Integration and Self-Efficacy): Review of progress, consolidation of learning, and development of post-group action plans, to build perceived self-efficacy.
- Session 12 (Existential factors and Termination): Reflection on group experience, acceptance of illness uncertainty, mutual peer support, and closure, to sustain gains post-intervention.

Therapy flow: 10 min warm-up → 60 min themed discussion → 20 min summary and homework assignment.

#### 2.3.3 Control group

**Figure 1:**
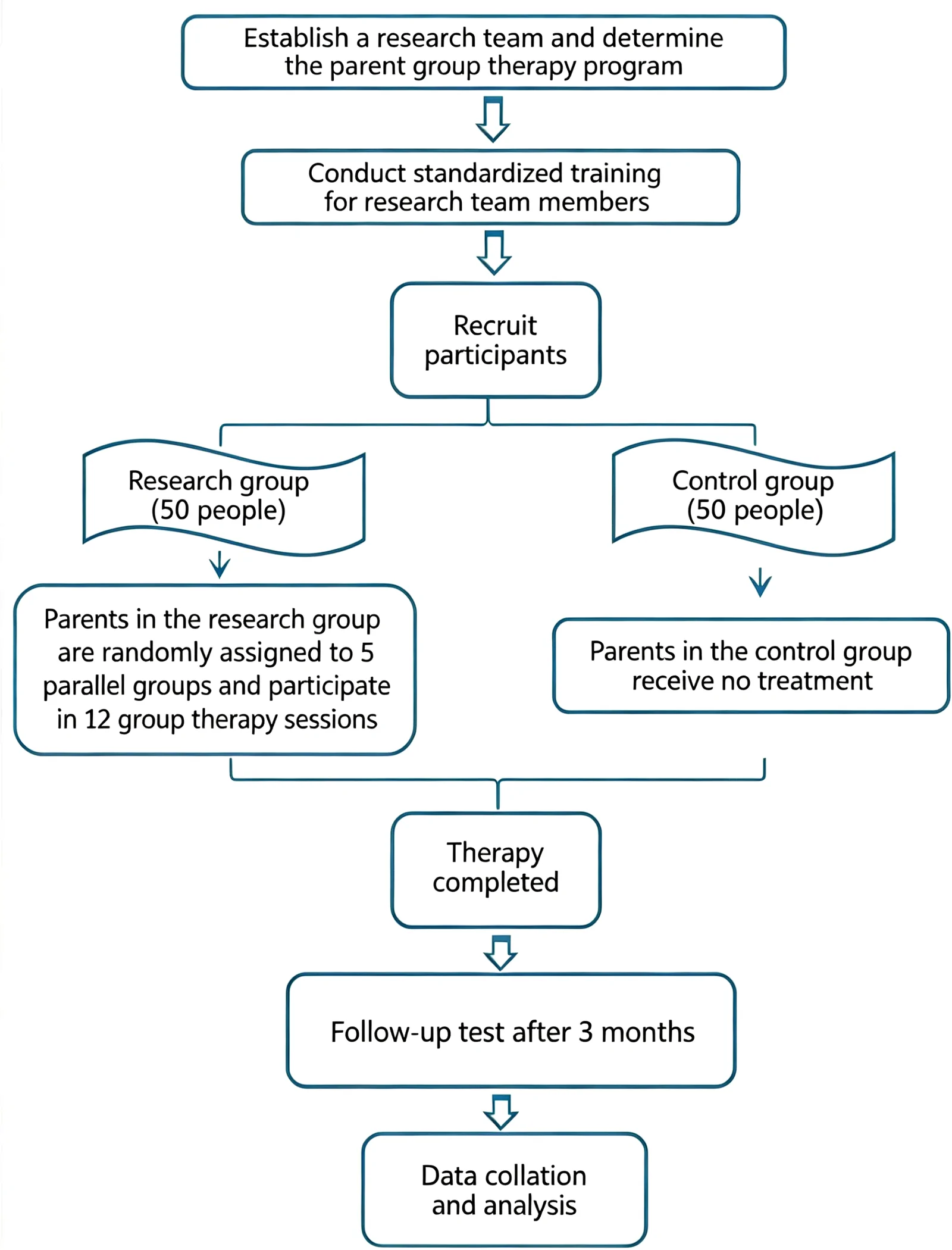
Research design flow chart.

Routine outpatient follow-up only; delayed group intervention was offered to all control group participants after completion of the 3-month follow-up assessment. The study design and participant flow are illustrated in **Figure 1**.

### 2.4 Measures

Sociodemographic questionnaire:

Parental age, gender, educational level, marital status, household income; adolescent illness duration, age, and gender.

The Self-Rating Anxiety Scale (SAS), developed by Zung (32), is a 20-item self-report instrument using a 4-point Likert scale, with higher scores indicating more severe anxiety symptoms. The Chinese version of SAS has been validated in Chinese community populations, with a Cronbach’s α coefficient of 0.733 reported in a recent large-sample study (33).

The Self-Rating Depression Scale (SDS), developed by Zung (34), is a 20-item self-report measure of depressive symptoms using a 4-point Likert scale, with higher scores indicating greater depression severity. The Chinese version of SDS has demonstrated acceptable internal consistency in Chinese rural female populations, with a Cronbach’s α coefficient of 0.784 reported in a previous validation study (35).

The Family Adaptability and Cohesion Scale II (FACES II) is a 30-item self-report measure consisting of two subscales: cohesion and adaptability. Cohesion reflects emotional bonding among family members, while adaptability refers to family flexibility in problem-solving and role adjustment; higher scores indicate better family functioning. The Chinese version (FACES II-CV), revised by Phillips et al., has shown good internal consistency, with Cronbach’s α coefficients of 0.83 for the cohesion subscale and 0.83 for the adaptability subscale (36).

### 2.5 Statistical analysis

SPSS 29.0 was used for all analyses. Continuous data are presented as mean ± SD. Baseline comparisons were conducted using independent t-tests for continuous variables and chi-square tests for categorical variables.

A 2×3 mixed-design repeated-measures ANOVA was applied to test group, time, and group-by-time interaction effects. Mauchly’s test of sphericity was conducted; the Greenhouse–Geisser correction was applied when the sphericity assumption was violated. *Post hoc* pairwise comparisons were performed with Bonferroni correction.

Partial eta-squared (*η*²) is reported as the effect size for ANOVA effects, with 0.01, 0.06, and 0.14 interpreted as small, medium, and large effects, respectively. For pairwise comparisons, 95% confidence intervals (*CIs*) for mean differences are provided.

The primary analysis used a completer sample. Missing data due to loss to follow-up were not imputed. As a sensitivity analysis, linear mixed-effects models (*LMMs*) with random intercepts for families were conducted to account for within-family clustering of parental data, to evaluate the robustness of the primary findings.

A *P*-value < 0.05 was considered statistically significant.

## 3 Results

### 3.1 Baseline characteristics

A total of 90 parents of adolescents with depression were included in the final analysis: 50 in the intervention group and 40 in the control group.

Baseline demographic and clinical characteristics were comparable between groups. No significant differences were found in parental age (46.02 ± 4.41 years vs. 45.90 ± 4.45 years), gender (50% female vs. 50% female), educational level, marital status, or adolescent illness duration, age (15.36 ± 2.50 years vs. 15.40 ± 1.67 years), and gender (64% girl vs. 60% girl) (all P > 0.05).

There were also no significant between-group differences in baseline SAS, SDS, or FACES II scores (all P > 0.05), confirming that the two groups were comparable at baseline.

### 3.2 Feasibility and retention

The overall retention rate was 90% at the 3-month follow-up (100% in the intervention group, 80% in the control group). For the intervention group, the mean number of sessions attended was 10.2 ± 1.8, corresponding to an 85.0% attendance rate. No adverse events related to the group intervention were reported during the study period.

### 3.3 Descriptive statistics of scale scores

SAS, SDS, family cohesion, and family adaptability scores at pre-intervention, post-intervention, and 3-month follow-up are presented in **Table 1**.

**Table 1:** Scale scores of anxiety, depression, family cohesion and adaptability in the two groups at different time points ( $\bar{\text{x}}$ ± s).

Measure	Group	Pre-intervention	Post-intervention	3-month follow-up
Anxiety (SAS)	Intervention	36.44 ± 8.91	35.12 ± 7.68	34.28 ± 7.85
	Control	35.70 ± 6.89	35.10 ± 7.30	35.50 ± 7.25
Depression (SDS)	Intervention	43.72 ± 10.06	43.48 ± 10.67	42.28 ± 10.77
	Control	44.10 ± 8.54	44.08 ± 8.41	44.15 ± 8.29
Family Cohesion	Intervention	83.14 ± 9.25	84.88 ± 8.56	86.46 ± 8.13
	Control	86.88 ± 9.28	87.02 ± 9.43	86.98 ± 9.40
Family Adaptability	Intervention	56.12 ± 8.63	58.10 ± 8.96	59.42 ± 8.24
	Control	57.55 ± 9.88	57.93 ± 9.68	57.88 ± 9.72

Intervention group, n=50; Control group, n=40.

**Figure 2:**
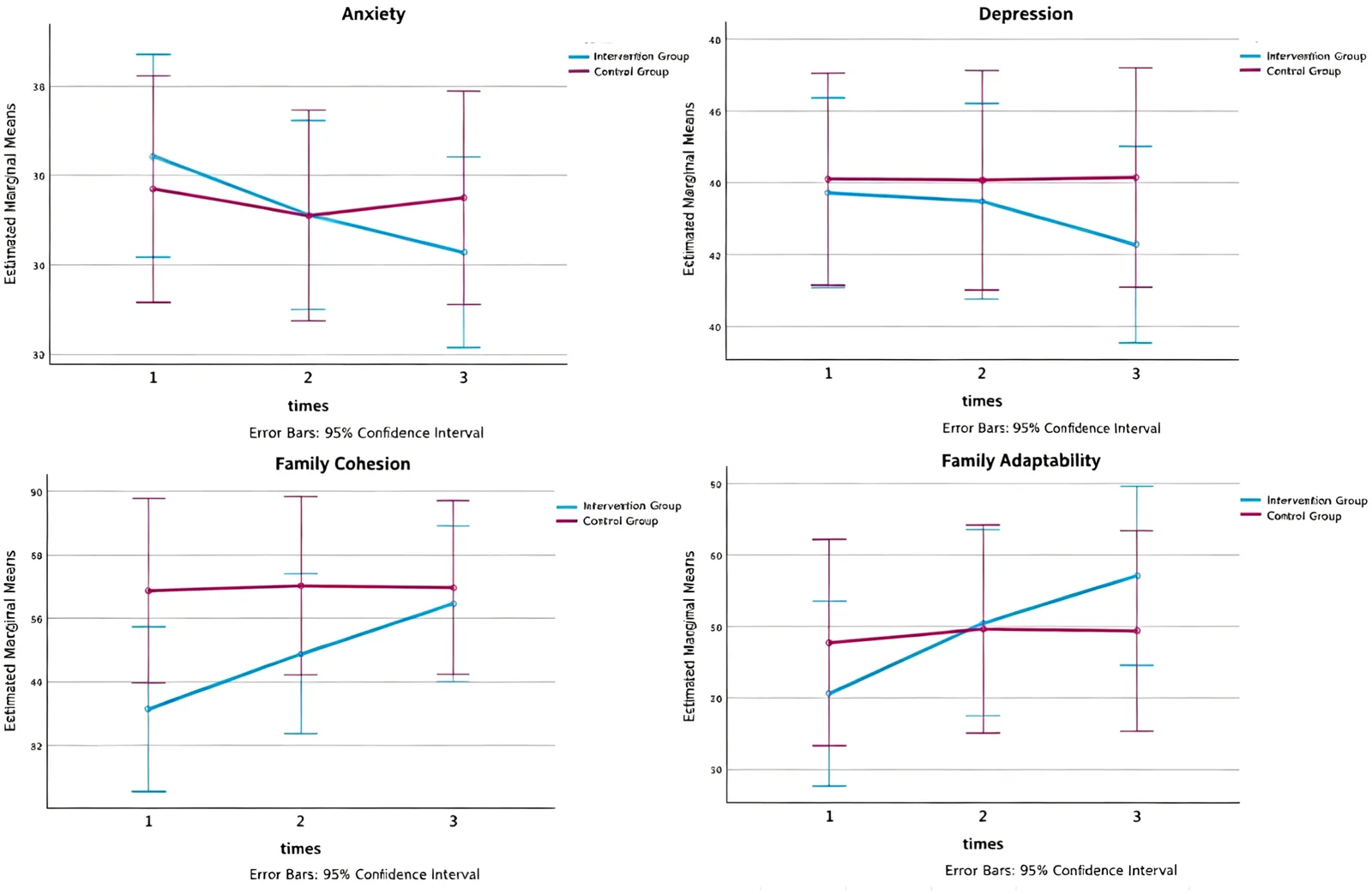
Longitudinal trends of scale scores in the two groups. The figure presents the longitudinal changes in four indicators: anxiety, depression, family cohesion, and family adaptability. The horizontal axis indicates measurement time points (pre-intervention, post-intervention, 3-month follow-up), and the vertical axis represents scale scores.

Anxiety scores in the intervention group decreased continuously over time, while family cohesion and adaptability increased steadily; No statistically significant changes were observed in the control group across all outcomes. Longitudinal trends for all indicators are illustrated in **Figure 2**.

### 3.4 Test of sphericity

Mauchly’s test of sphericity: Anxiety *W* = 0.873, *χ²* = 11.799, *P* = 0.003; Depression *W* = 0.924, *χ²* = 6.884, *P* = 0.032; Cohesion *W* = 0.790, *χ²* = 20.550, *P* < 0.001; Adaptability *W* = 0.824, *χ²* = 16.815, *P* < 0.001. The sphericity assumption was violated for all outcomes; therefore, the Greenhouse–Geisser correction was applied in all subsequent repeated-measures analyses.

### 3.5 Results of repeated-measures ANOVA

#### 3.5.1 Main effect of group

Between-subjects effects testing showed no significant overall between-group differences in parental anxiety, depression, family cohesion, or family adaptability when averaged across time points (all *P* > 0.05), consistent with baseline equivalence.

#### 3.5.2 Main effect of time

After Greenhouse–Geisser correction, significant main effects of time were found for anxiety (*F* = 3.516, *P* = 0.037, *η²* = 0.038), family cohesion (*F* = 5.080, *P* = 0.011, *η²* = 0.055), and family adaptability (*F* = 3.930, *P* = 0.028, *η²* = 0.043). The main effect of time for depression was not significant (*F* = 0.637, *P* = 0.519, *η²* = 0.007).

Pairwise comparisons showed that anxiety scores at the 3-month follow-up were significantly lower than at baseline (mean difference = −1.18, 95% *CI* [−1.98, −0.38], *P* = 0.004). Family cohesion and adaptability scores at follow-up were significantly higher than at baseline (cohesion: mean difference = 1.71, 95% *CI* [0.61, 2.81], *P* = 0.003; adaptability: mean difference = 1.81, 95% *CI* [0.57, 3.05], *P* = 0.005).

#### 3.5.3 Group × time interaction effect

A significant group-by-time interaction was observed for family cohesion (*F* = 4.487, *P* = 0.018, *η²* = 0.049), indicating differential change trajectories between groups. For family adaptability, the group-by-time interaction showed a nonsignificant trend (*F* = 2.576, *P* = 0.088, *η²* = 0.028). No significant group-by-time interactions were found for anxiety (*F* = 2.194, *P* = 0.121, *η²* = 0.024) or depression (*F* = 0.758, *P* = 0.461, *η²* = 0.009).

Further linear trend interaction analysis revealed significant between-group differences in the linear trajectories of anxiety, family cohesion, and adaptability (all *P* < 0.05). Sensitivity analyses using linear mixed-effects models accounting for within-family clustering yielded results consistent with the primary *ANOVA* analyses, with no substantive changes in the significance of interaction effects.

Longitudinal changes in outcome measures are illustrated in **Figure 2**.

## 4 Discussion

### 4.1 Effects of the psychoeducational group intervention on parental anxiety

The onset and diagnosis of adolescent depression impose persistent psychological stress on caregivers. Long-term caregiving burden, uncertainty about illness trajectory, parent–child communication difficulties, and mental health stigma easily trigger anxiety and distress, creating a vicious cycle of adolescent depression → family stress → impaired family functioning.

Previous studies have shown that group counseling can enhance resilience and hope, and reduce anxiety and depression among parents of hospitalized adolescents with depression (25). A 12-week parenting group was also found to improve parental mentalization and family functioning while reducing harsh parenting practices (30). The core therapeutic principles of Yalom’s interpersonal theory—including interpersonal interaction, peer support, emotional catharsis, and existential reflection (37). Although these therapeutic processes were not directly measured in the present study, they provide plausible explanatory mechanisms for the observed trends.

Our results showed that anxiety scores in the intervention group decreased steadily over the study period, with effects sustained at the 3-month follow-up. However, the group-by-time interaction for anxiety was not statistically significant, meaning we cannot conclude that the reduction was specifically attributable to the intervention. No significant changes in anxiety were observed in the control group.

### 4.2 Value of the structured psychoeducational group intervention in optimizing family function

Family systems theory posits that adolescent depression is not merely an individual psychological problem, but an external manifestation of imbalanced family interaction patterns, weak emotional bonds, and insufficient family coping capacity. Mian et al. (38) noted that family type and parenting styles are associated with depressive symptoms among middle school students. As children’s “first teachers, “ parents’ parenting styles are closely related to their children’s physical and mental health (39–42). Parental control, a common parenting practice, is not only associated with child and adolescent depressive symptoms but may also contribute to the development of depressive personality traits (43), and this association has been documented across multiple countries (44–47), indicating that parenting has a substantial impact on adolescent depression. Meanwhile, healthy family functioning serves as a core protective factor for adolescent recovery from depression (23).

The present study found that family cohesion and adaptability among parents in the intervention group improved steadily throughout the intervention, with a significant group-by-time interaction effect for family cohesion. This confirms that the intervention showed preliminary benefits for family cohesion, with effects sustained over 3 months.

A key strength of this study, distinguishing it from previous research, is that both parents of adolescents with depression participated in the same group, maximizing the efficiency of parent support. During the intervention, parents developed awareness of the negative impacts of maladaptive parenting and communication patterns through self-reflection and group feedback, and proactively adjusted family interactions. The group also provided positive role models, helping parents learn evidence-based strategies for communication, emotion regulation, and family problem-solving, thereby enhancing family resilience in the face of mental illness (30). Joint participation by both parents fosters marital collaboration, encouraging mutual reminders and intentional changes in daily family dynamics.

Family function in the control group remained unchanged throughout the study, further indicating that without professional psychological intervention and cognitive guidance, dysfunctional family systems are unlikely to repair spontaneously, highlighting the clinical value of parent-focused group therapy.

### 4.3 Limited effects on parental depressive mood

No significant intervention-specific improvement in parental depression was observed, which is inconsistent with previous findings by Qin et al. (25). Potential explanations include: First, study samples differed: Qin et al. recruited parents of adolescents hospitalized for depression, while the present study included outpatient parents, which may have influenced intervention responsiveness. Second, illness severity may differ: parents of inpatients may experience more severe distress and greater room for improvement, whereas outpatient parents in our sample had milder depressive symptoms at baseline. Additionally, depression is a persistent, internalized mood disorder shaped by stable factors such as personality traits, chronic stress, and long-standing maladaptive coping styles. Short-term group intervention can alleviate situational stress but may be insufficient to alter entrenched depressive tendencies.

### 4.4 Characteristics of intervention effects

No significant cross-sectional between-group differences were observed at any single time point. Intervention effects were primarily reflected in longitudinal changes over time, consistent with the gradual nature of psychological change. Shifts in cognition, emotion, and family relationships develop slowly and cumulatively; sustained intervention effects are better captured through long-term follow-up. The progressive improvement in the intervention group and stability in the control group suggest that the group therapy may have potential in preventing family function decline and negative emotion accumulation, promoting positive family development.

### 4.5 Study limitations

This study has several limitations. First, only a waitlist control group was included, without an active control condition such as routine health education or general psychological support groups. Therefore, we cannot compare the efficacy of this Yalom-principles-informed psychoeducational group intervention with other conventional interventions, nor can we definitively attribute observed changes to Yalom-specific therapeutic factors rather than nonspecific factors such as attention, peer support, or treatment expectation. Second, the follow-up period was limited to 3 months post-intervention, which is insufficient to observe the long-term stability, decay pattern, and sustained clinical benefits of the intervention. Third, the sample was recruited exclusively from outpatient clinics at a single hospital in Beijing, limiting the representativeness and generalizability of findings to other regions or clinical settings. Fourth, all outcome measures relied on self-report scales, which may be subject to response bias and social desirability effects. Future studies could combine subjective ratings with objective assessments or informant reports to validate intervention effects. Fifth, although sensitivity analyses accounting for within-family clustering produced consistent results, the primary ANOVA did not formally model the non-independence of data from couples within the same family, which may have slightly inflated Type I error rates. Sixth, this study did not explore potential moderators or mediating mechanisms of intervention effects, such as parental personality traits, family socioeconomic status, or adolescent illness severity; underlying mechanisms remain to be further investigated.

### 4.6 Future research directions

Future studies could address these limitations: (1) Conduct multi-center, large-scale trials with diverse samples to enhance generalizability. (2) Include active control groups to clarify the unique therapeutic effects of the the Yalom-principles-informed psychoeducational intervention. (3) Extend follow-up to 6–12 months to evaluate long-term outcomes. (4) Combine subjective scales with objective or multi-informant assessments to validate intervention effects. (5) Explore mediating and moderating mechanisms linking the intervention to parental emotion and family function. (6) Refine and standardize the intervention manual to support broader clinical dissemination.

## 5 Conclusion

A 12-session structured psychoeducational group therapy grounded in Yalom’s interpersonal therapeutic principles was feasible to implement among parents of adolescents with depression and showed preliminary evidence of improving family cohesion, with effects sustained for 3 months, with possible benefits for family adaptability and anxiety. However, intervention-specific effects on parental depression was not statistically significant. Further studies using active control conditions, larger samples, and analytic methods accounting for couple-level data are needed to confirm efficacy.

## Data Availability

The original contributions presented in the study are included in the article/supplementary material. Further inquiries can be directed to the corresponding authors.
